# When Manual Analysis of 12-Lead ECG Holter Plays a Critical Role in Discovering Unknown Patterns of Increased Arrhythmogenic Risk: A Case Report of a Patient Treated with Tamoxifen and Subsequent Pneumonia in COVID-19

**DOI:** 10.1007/s12012-021-09659-w

**Published:** 2021-05-20

**Authors:** Donatella Brisinda, Barbara Merico, Peter Fenici, Riccardo Fenici

**Affiliations:** 1 Fondazione Policlinico Universitario Agostino Gemelli-IRCCS, Università Cattolica del Sacro Cuore, Largo Agostino Gemelli 8, 00168 Rome, Italy; 2 Biomagnetism and Clinical Physiology International Center (BACPIC), Viale dell’Astronomia, 12, 00144 Rome, Italy

**Keywords:** Tamoxifen, Drug toxicity, Long QT syndromes, Ventricular repolarization reserve, Breast cancer, Exemestane

## Abstract

Several medicines, including cancer therapies, are known to alter the electrophysiological function of ventricular myocytes resulting in abnormal prolongation and dispersion of ventricular repolarization (quantified by multi-lead QTc measurement). This effect could be amplified by other concomitant factors (e.g., combination with other drugs affecting the QT, and/or electrolyte abnormalities, such as especially hypokalemia, hypomagnesaemia, and hypocalcemia). Usually, this condition results in higher risk of torsade de point and other life-threatening arrhythmias, related to unrecognized unpaired cardiac ventricular repolarization reserve (VRR). Being VRR a dynamic phenomenon, QT prolongation might often not be identified during the 10-s standard 12-lead ECG recording at rest, leaving the patient at increased risk for life-threatening event. We report the case of a 49-year woman, undergoing tamoxifen therapy for breast cancer, which alteration of ventricular repolarization reserve, persisting also after correction of concomitant recurrent hypokalemia, was evidenced only after manual measurements of the corrected QT (QTc) interval from selected intervals of the 12-lead ECG Holter monitoring. This otherwise missed finding was fundamental to drive the discontinuation of tamoxifen, shifting to another “safer” therapeutic option, and to avoid the use of potentially arrhythmogenic antibiotics when treating a bilateral pneumonia in recent COVID-19.

**Supplementary Information:**

The online version contains supplementary material available at 10.1007/s12012-021-09659-w.

## Introduction

Drug-induced prolongation of ventricular repolarization is the most common and preventable cause of the so-called secondary Long QT syndrome (LQTS), which may imply high risk of life-threatening ventricular arrhythmic events [1–3]. Other concomitant factors, such as comorbidities, abnormal drug’s metabolism due to CYP2D6 genes polymorphism [4], structural cardiac disorders, electrolyte abnormalities, repolarization heterogeneity, autonomic nervous system imbalance, age, sex and even sleep’s phases may also affect the QT duration, especially in patients with unrecognized/unknown ion channel genes polymorphism and reduced ventricular repolarization reserve (VRR) [5, 6].

Breast cancer is estimated to accounts for 13.3% of all new cancer cases diagnosed in EU-27 countries in 2020, making it the most frequently occurring cancer, accounts for 28.7% of all new cancers in women [https://ecis.jrc.ec.europa.eu/pdf/Breast_cancer_factsheet-Dec_2020.pdf] and it is the most frequently diagnosed cancer among US women too [7]. Treatment strategies are nowadays chosen based on the type of cancer, size and stage of the disease, presence of estrogen or progesterone receptors, or other phenotypes. Whenever possible, surgery represents a first-line therapeutic option, often associated with radiotherapy and chemotherapy, based on hormone therapy (selective estrogen receptors modulation as tamoxifen or aromatase-inhibitors as anastrozole), target therapy (for breast cancer with high level of protein HER2) and immunotherapy (atezolizumab and sacituzumab for triple-negative breast cancer) [8].

Tamoxifen is an estrogen receptor antagonist commonly used to treat patients whose breast cancer are estrogen receptor-positive [9], and has been shown to experimentally prolong QT interval duration by direct inhibition of rapidly activating delayed rectifier potassium (IKr) channels and clinically by affecting cytochrome enzymes that metabolize tamoxifen, one of which is the CYP 3A4 enzyme [10–12]. Since tamoxifen treatment is usually needed for several years, it is likely that other comorbidities might either co-exist or develop over time, requiring concomitant treatments inducing drug-to-drug interactions, producing abnormal structural and electrophysiological substrates, increasing arrhythmogenic risk.

Therefore, breast cancer patients treated with tamoxifen must be closely monitored with periodical ECG recordings and assessment of the heart rate (HR) corrected calculation of the QT duration (QTc) according to standardized methods [13–16]. Since there are no standards for interpreting prolonged QTc intervals from Holter monitoring records, QTc assessment from ECG ambulatory monitoring is usually not recommended [4]. However, as occurred in the reported case, the QTc interval automatically calculated by computer-assisted analysis of standard 10-s ECGs at rest could be unable to evidence dynamic abnormality of the QTc interval, in patients with unknown reduced repolarization reserve.

The reported case demonstrates how the careful assessment of relevant information from the patient’s medical history and ongoing treatments, triggered an “a-priori suspect of possible LQTS” and prompted the consequent manual measurements of QTc from 12-lead Holter ECG, unveiling the unknown increased arrhythmogenic risk that could have most probably be precipitated by the subsequent anticipated administration of azithromycin or quinolones for the treatment of the bilateral pneumonia complication in COVID-19.

## Case Report

We report a 49-year-old woman, without family history or previous known comorbidities (other than hypothyroidism under hormonal supplementation), who was diagnosed a single lesion on the upper-outer quadrant of left breast at a Rx mammography and echo routine screening visit, on October 2019.

**Fig. 1:**
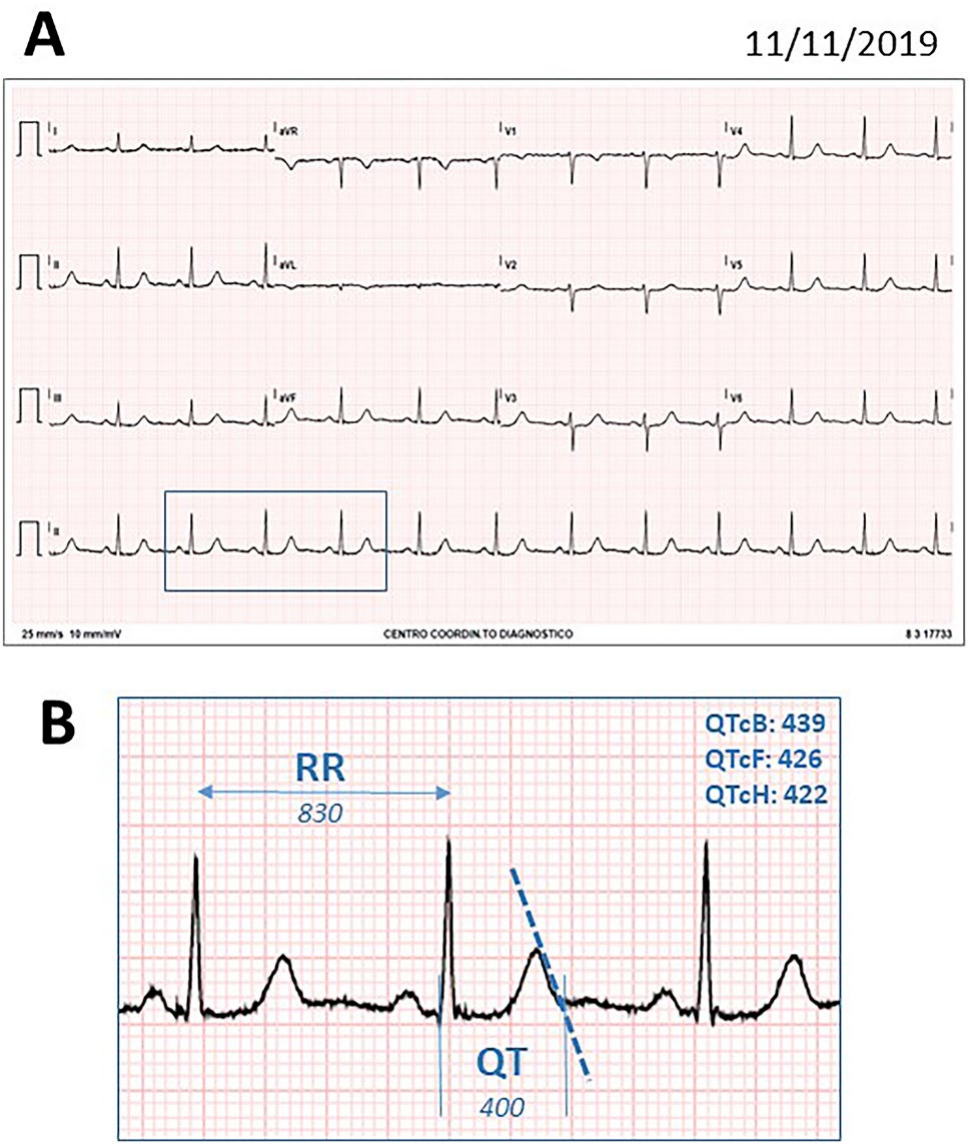
**a** Normal baseline pre-surgery standard ECG recording. **b** The QT interval was measured from the beginning of the QRS complex to the end of T-wave (defined by the intersection point between the tangent drawn at the maximum downslope of the T-wave and the iso-electric line), and normalized for heart rate changes as QTc using the Bazett (QTcB), Fridericia (QTcF) and Hodges (QTcH) formulas, as appropriate [13]

On November 2019, according to the tumor board decision, she underwent quadrantectomy with sentinel lymph node biopsy. Histology confirmed ductal carcinoma in situ (pTis, pN0 (0/1)-ER 90%, PR 60%, HER2 1+). Thus, adjuvant radiotherapy for 3 months (from December 2019 to February 2020) and hormone therapy (tamoxifen 20 mg/day and leuprorelin acetate 3.75 mg/month) were performed. Pre-surgery standard 12-lead ECG was normal (Fig. 1).

**Fig. 2:**
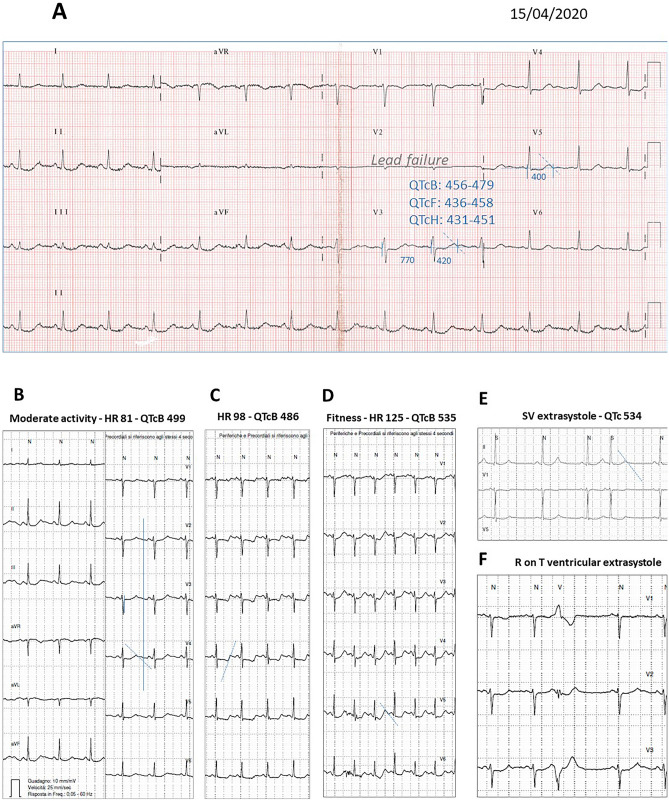
**a** Standard 12-lead ECG during Tamoxifen treatment and hypokalemia (K+ plasma level 3.3 mmol/L). First 12-lead Holter monitoring: evidencing clear-cut prolongation of the QTc interval (**b**), and precordial VR alteration during phases of moderate (**c**) or intense (**d**) physical activity or sudden shortening of the cycle length (**e**), due to a supraventricular premature beat. **f** Isolated R on T ventricular extrasystole originating in the left ventricle and compatible with triggered activity

On April 15th 2020, after about 5 months of tamoxifen treatment, she has been referred to a cardiology consultation due to symptoms of palpitation. The physical examination was normal, standard 12-lead ECG at rest showed aspecific ventricular repolarization (VR) abnormality, with prominent U-wave in leads V3–V5 in the presence of moderate hypokalemia (K+ plasma level was 3.3 mmol/L). Since the Bazett and Fridericia formulas have been reported to overestimate the change in QT interval when heart rate increases, we have also calculated the Hodges one [13]. From the standard rest ECG, the QTc appeared slightly prolonged if calculated according to the Bazett (QTcB between 456 and 479 ms) and the Fridericia (QTcF) formulas, but it was within normal female limits with Hodges (QTcH) one, with some dispersion in different leads (Fig. 2a).

12-lead ECG Holter monitoring confirmed borderline average rest values of QTc (QTcB 470 ms). However, phases of clear-cut QTc interval prolongation (QTc B: 595 ms at wake-up, and even above 500 ms also with QTcH correction—see Table 1 in supplementary information) were detected during heart rate increase induced by moderate or intense physical activity, during REM sleep, and after sudden cycle length shortening due to supraventricular extrasystoles. Isolate ventricular extrasystoles were also recorded, compatible with early after depolarization mechanism (Fig. 2b–f).

We hypothesized that the QTc prolongation could be due to a side effects of tamoxifen in concomitance with hypokalemia, and oral potassium supplement and antialdosteronic therapy were started.

**Fig. 3:**
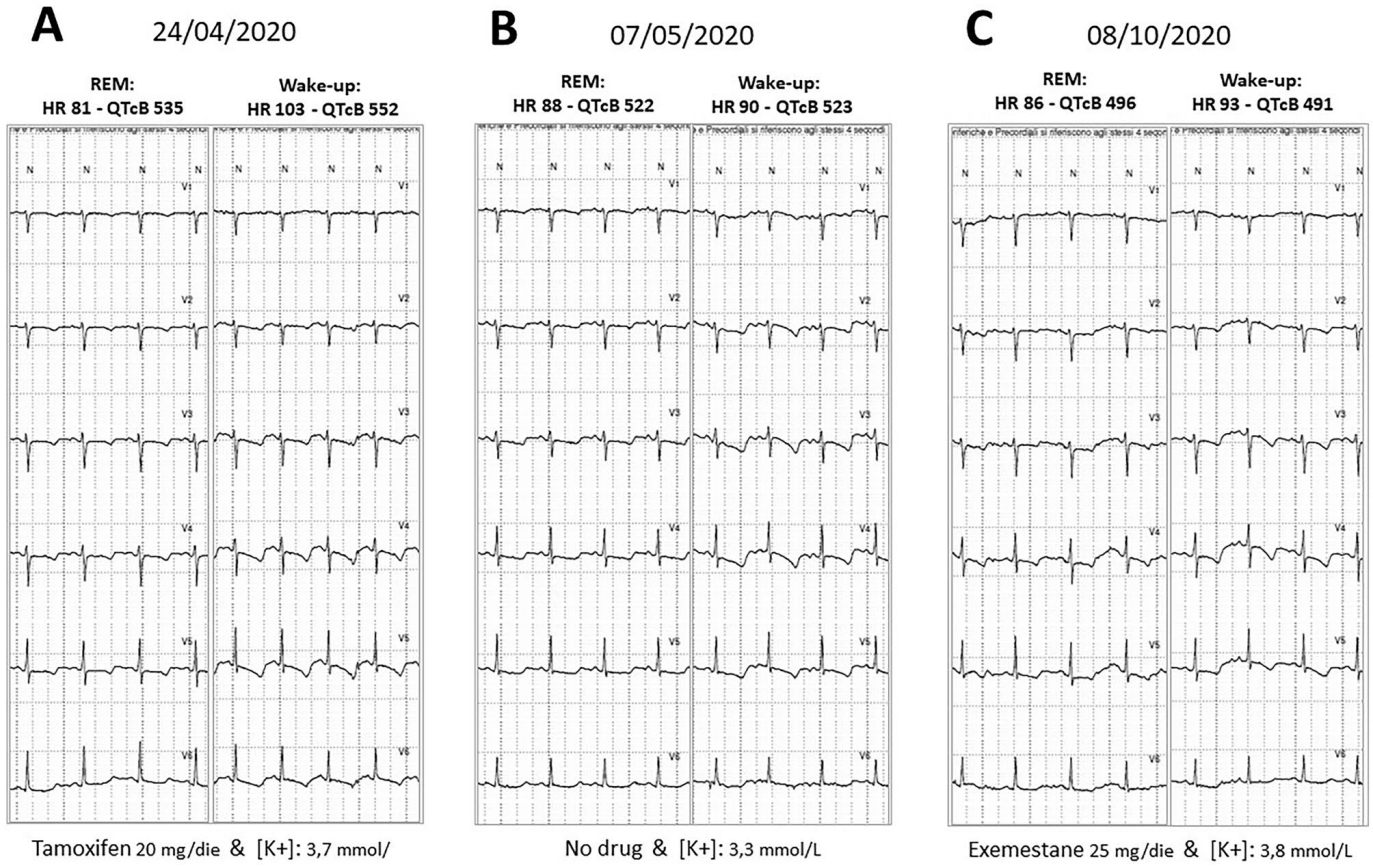
**a** Tamoxifen and normal [K+] plasma level. Progressive shortening of the QTc after tamoxifen discontinuation in spite of recurrent hypokalemia (**b**), and during treatment with exemestane 25 mg/die (**c**). Persistence of transient precordial ST–T-wave alteration during phases of sympathetic activation (REM sleep and Wake-up)

However, after 1 week (April, 24th), in spite of K+ plasma level normalization (3.7 mmol/L) and normal rest ECG, a second 12-lead Holter monitoring evidenced the persistence of clear-cut QTc prolongation (at wake-up, QTc B still above 500 ms, QTc F and QTc H about 500 ms, see Table 1 in supplementary information) and of VR abnormalities (negative ST shift with asymmetric negative T-wave in V2–V5,) during phases of sympathetic activation and HR increment induced by moderate physical activity, during REM sleep and at wake-up (Fig. 3a).

Genetic test excluded congenital LQT syndromes. Treadmill effort test, transthoracic echocardiogram, adrenal hormones, and renal function were normal (eGFR: 78 ml/min). The urine electrolytes concentration was within normal range. The patient was asymptomatic for angina and VR abnormalities were partially improved during cardiofitness training as previously shown (Fig. 2d).

Although the oncologist would have preferred to continue tamoxifen implying fewer side effects in a female patient still in fertile age, hypothesizing a background impaired VRR [5, 6] as the potential cause of tamoxifen-induced QTc prolongation, that drug was discontinued in agreement with the oncologist on April 30th, while leuprorelin acetate was continued because it may only rarely affect the QT duration.

One week later (May 7th), rest QTc was within normal limits (see Fig. 1, in the supplementary information), in spite of recurrent hypokalemia (K+ plasma level 3.3 mmol/l—the patient had self-discontinued potassium supplement and antialdosteronic therapy), but transient phases of QTc prolongation (up to 523 ms) were still appreciable at 12-lead Holter monitoring, which in addition evidenced persistence of other VR abnormalities (Fig. 3b).

After K+ plasma level normalization with oral potassium supplement and antialdosteronic therapy, hormone therapy with an aromatase-inhibitor (exemestane 25 mg/die) was initiated.

The patient remained asymptomatic during the subsequent follow-up. On October 8th 2020, average values of QTc were within normal limits (QTcB: 465; QTcF: 441; QTcH: 440 ms) at the 12-lead Holter monitoring, although transiently prolonged values (now below 500 ms) and VR abnormalities were still appreciable during phases of sympathetic activation (Fig. 3c and supplementary Table 2C).

In summary, retrospective systematic analysis of the four Holter recordings including comparative assessment during different rest and stress conditions, NREM and REM sleep (Table 1 in supplementary information), confirmed the following:

1. Rest QTc values automatically measured from the standard 10-s of 12-lead ECG were always within normal limits (or borderline in the presence of hypokalemia).
2. Corresponding 12-lead Holter recordings evidenced clear-cut VR abnormalities with prolongation of QTc values (calculated with three normalization methods), during phases of sympathetic enhancement, especially at wake-up, and HR increase (range of QTcB: 499–595 ms; QTcF: 475–542 ms; QTcH: 484–528), (Table 1 in supplementary information).
3. Highest QTc values were measured during concomitant tamoxifen treatment and hypokalemia.
4. After tamoxifen discontinuation a progressive shortening of average QTc values was observed (Table 2A in supplementary information), which was statistically significant (*p* < 0.05) for QTcH even in the presence of hypokalemia (Table 2B in supplementary information).
5. Under normal [K+] plasma level, all QTc values were significantly (*p* < 0.05) longer under tamoxifen treatment compared with those under exemestane (all within normal range) (Table 2C in supplementary information).
6. Comparative sub analysis of QTc values among rest and activity conditions confirmed that tamoxifen-induced impairment of VRR (i.e., QTc prolongation) was statistically significant only during phases of sympathetic activation.

**Fig. 4:**
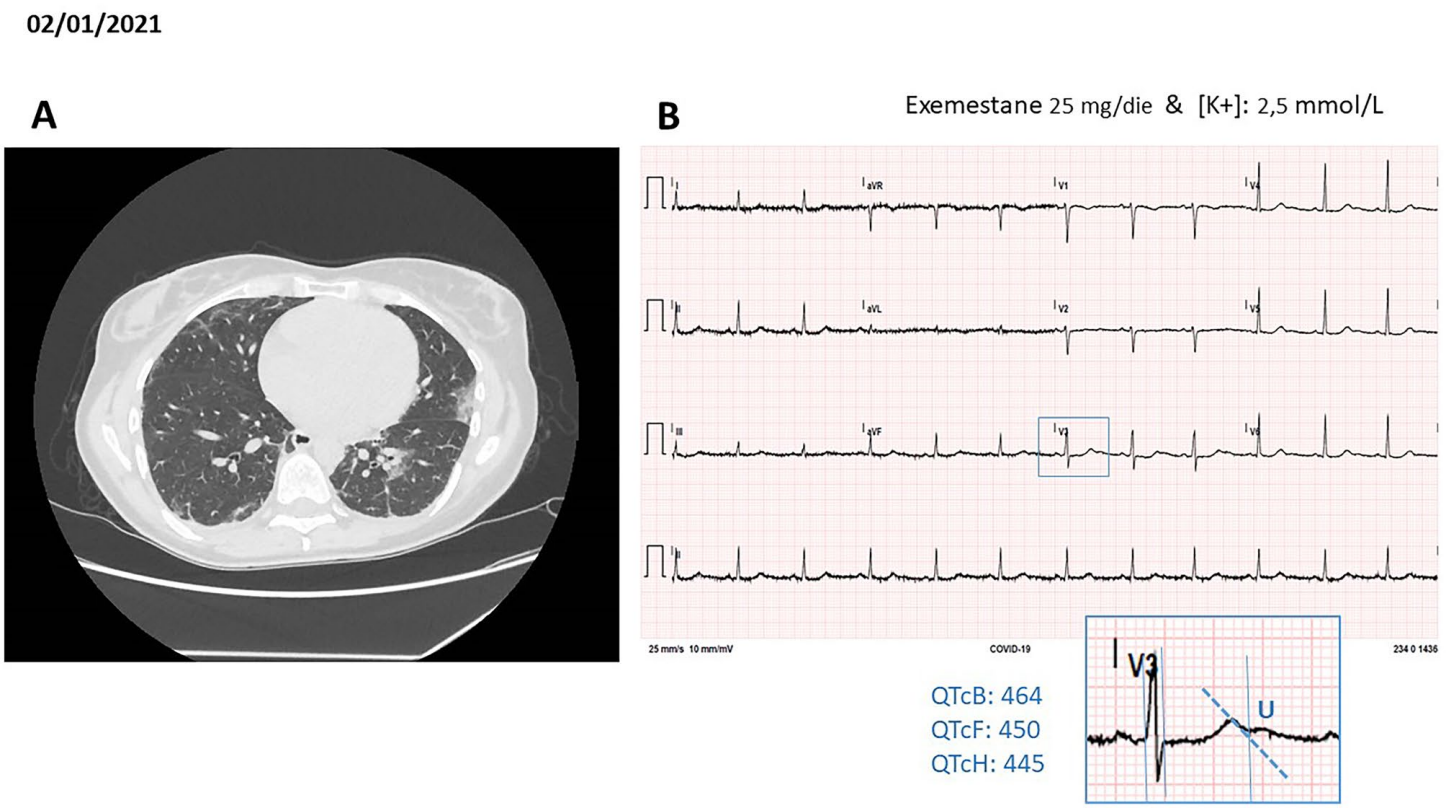
COVID-19 pneumonia. **a** Pulmonary TC scan with evidence of bilateral pneumonia. **b** Rest standard ECG with evidence of wide hypokalemia-related U-wave ([K+] 2.5 mmol/L), but QTc yet within normal limits

At the end of December 2020, the patient was hospitalized for COVID-19 complicated by bilateral pneumonia (Fig. 4a) and diarrhea-related hypokalemia (2.5 mmol/L). 12-lead ECG was within normal limits (QTc 445–460 ms), but with distinct U-wave in V3–V6 leads (Fig. 4b).

Because of the “a-priori” knowledge of impaired VR reserve, azithromycin and quinolones were avoided, and pneumonia was successfully treated with amoxicillin–clavulanic acid fixed dose combination. Hypokalemia was corrected with polarized KCl infusion until normalization.

No significant QTc prolongation nor arrhythmia were observed during the hospitalization.

## Discussion

Since its earliest definition, in 1998, the concept of “cardiac ventricular repolarization reserve” suggests that in the normal hearts there are complex homeostatic mechanisms to guarantee rapid and ordered VR and to avoid the risk of arrhythmogenic re-entrant circuits or triggered activity [5, 6]. Such safety margin is provided by a sort of functional integration and some redundancy of multiple repolarization currents that adaptively interact to compensate eventual dysfunction of one component [5]. However, if normal VRR is reduced, congenitally and/or as a consequence of pharmacological side effects, or of inflammation and cytotoxic injuries, the risk of life-threatening ventricular arrhythmias and even sudden death may be enhanced [5, 6, 15, 17, 18]. The most frequent indicator of such risk is the QTc prolongation. More than 13 genes [1] have been associated with congenital LQTs, but less is known about possible genetic etiology of subclinical dysfunction of voltage-gated channels and impaired VRR [5].

According to the National Cancer Institute (NCI) 4 degrees of QT prolongation induced by anticancer drugs must be considered, being: grade 1 (QTc 450–480 ms); grade 2 (QTc 481–500 ms); grade 3 (QTc > 501 ms on at least two ECG); grade 4 (QTc > 501 ms or a change of > 60 ms from baseline and torsade de point, polymorphic ventricular tachycardia, or signs or symptoms of severe arrhythmia)*.* Grades 3 and 4 are considered potential risk of life-threatening events [18].

Among other cancer drugs, tamoxifen may induce QT interval prolongation and *torsade de point*, by itself or when other conditions affecting the VRR (for example electrolyte abnormalities or concomitant drugs prescription) may overlap at same time [3, 10–12]. Thus prolonged tamoxifen treatment is usually monitored with periodical standard 12-lead ECG to exclude drug-induced side effects on ventricular repolarization [5, 12]. Although this approach is considered sufficiently sensitive, and Holter monitoring of QTc interval duration is usually not recommended [4] because the complexity of the physiological control of circadian modulation of ventricular repolarization rate dependence has not been yet fully clarified and the range of normality and reproducibility of QTc values under dynamic conditions (as measurable from Holter monitoring) are still uncertain [19–21], we found that it was useful to discover otherwise unknown dynamic abnormality of the QTc interval, suggestive of impaired VRR, primarily determined by tamoxifen and requiring its discontinuation, although the oncologist favored that drug as first choice.

The lack of normality ranges of circadian QTc values measured from Holter monitoring could be a limitation in interpreting our data. However, although peak values of QTc close to 500 ms (shortly after awakening), have been individually observed [19], the peak QTc values at wake-up were much higher in our reported case during tamoxifen treatment (initial QTc Bazett: almost 600 ms), thus undoubtedly abnormal. Moreover, the follow-up recordings demonstrate that after discontinuation of tamoxifen peak QTc values at wake-up became significantly shorter (independently of potassium plasma levels) and remained within normal limits also during exemestane treatment.

Since such abnormality was not evident at the standard 10-s 12-lead ECG recording, this, in our opinion, is a reasonable proof that in the reported case the evaluation of QTc variability provided by Holter monitoring was pivotal to discover an unknown and potentially arrhythmogenic reduction of VRR, likely enhanced by tamoxifen treatment, which for safety had to be withdrawn.

Moreover, such observation was useful not only to guide the selection of a more appropriate cancer treatment, but also to prevent the contraindicated and potentially arrhythmogenic administration of azithromycin or quinolones when the patient needed additional treatment for bilateral pneumonia in COVID-19.

As concerns the transient and asymptomatic ST and T-wave abnormalities observed during phases of sympathetic activation, their mechanistic interpretation is more difficult. An ischemic origin was reasonably ruled out by the absolute absence of angina symptomatology, their disappearance during maximum physical effort and by the asymmetric morphology of the negative T-waves. Being the patient asymptomatic, nuclear perfusion imaging scan was not performed, to avoid unnecessary additional irradiation. Theoretically it could be hypothesized that subclinical radiotherapy-induced myocardial fibrosis [22], sparsely affecting also the peripheral cardiac autonomic innervation [23], could alter the local synchronism of ventricular depolarization and secondarily of the repolarization wavefronts during sudden changes of the sympathetic drive [24].

Also, the cause of recurrent hypokalemia remains unclear, being renal and adrenal functions within normal range one could hypothesize a defect of gastrointestinal absorption, but that possibility has not been ascertained so far.

## Conclusion

The reported case confirms that before starting anticancer treatment, especially in female patients, cardiac ventricular repolarization reserve should be preliminarily assessed to prevent potentially arrhythmogenic drug-induced abnormal prolongation of the QTc interval and risk of life-threating arrhythmias. Whereas other methods can be somehow difficult to perform or ethically questionable [5], periodical 12-lead Holter recording was sensitive enough to evidence and monitor QT and T-wave abnormalities, whereas the 10-s standard 12-lead ECG at rest was apparently normal or showed aspecific VR alteration only.

Luckily in the reported case, although the QTc prolongation frequently exceeded 500 ms, major high-risk LQT syndromes were ruled out by the negative results of genetic tests and only rare single premature ventricular beats were observed. This suggests that, although impaired and generating transient ECG abnormality, the patient’s VRR was still sufficient to prevent the occurrence of dangerous ventricular arrhythmias and that probably a minor ion channel dysfunction has been unmasked only eventually by the overlapping of external factors such as tamoxifen-induced block of the IKr, enhanced by recurrent hypokalemia and only partially compensated by a gain of function of the IKs channel [5].

Nevertheless, it was also evidenced that the recording of 10-s standard 12-lead ECG at rest [17], may be insufficiently sensitive to identify and to safely monitor patients with unknown alteration of physiologic VRR [5, 6].

Therefore, we suggest that a baseline 12-lead ECG Holter monitoring, with accurate manual measurement of QTc, especially during sudden changes of HR under different day and night conditions, should be routinely performed at least before starting tamoxifen treatment [12].

The cost–benefit ratio of follow-up Holter monitoring can change on the basis of local reimbursement policy. However, costs may be reduced with nowadays sensor and telematic transmission technologies and could favor the design of a randomized study as the next step, helpful to investigate the complexity of the physiological control of circadian modulation of ventricular repolarization rate dependence and to better define the normality range of QTc dynamics on larger populations [25].

This is even more important nowadays, when the unpredictable occurrence of COVID-19 related complications may multiply the risk of arrhythmogenic events due to direct myocardial injury induced by the coronavirus and/or potential cardiotoxicity of drugs used to treat the cases with severe respiratory and cardiovascular complications, such as quinolone antibiotics, azithromycin, and antiretroviral therapies that may enhance the risk of adverse interactions and electrical instability increasing QT prolongation and transmural heterogeneity of ventricular repolarization. Such arrhythmogenic mechanisms can be enhanced by hypokalemia, cytokines-induced hyperactivation of cardiac sympathetic system, IL-6-induced inhibition of HERG potassium channels with consequent prolongation of ventricular action potentials and IL-6-induced inhibition of cytochrome P450 (CYP) 3A4 which may increase the bioavailability of the above mentioned QT-prolonging medications [26].

## Supplementary Information

Below is the link to the electronic supplementary material.

Supplementary file1 (DOCX 354 kb)

## Data Availability

A Table with raw QTc data is available in the supplementary information. Additional data (Standard ECG and Holter recordings, output of SPSS statistics) might be available on request after de-identification, in compliance with applicable privacy laws, data protection and requirements for consent and anonymization.
