# Supplementary material for: When Manual Analysis of 12-Lead ECG Holter Plays a Critical Role in Discovering Unknown Patterns of Increased Arrhythmogenic Risk: A Case Report of a Patient Treated with Tamoxifen and Subsequent Pneumonia in COVID-19
Source: Cardiovasc Toxicol. 2021 May 20;21(9):687–94. doi: 10.1007/s12012-021-09659-w (PMC8136377; doi:10.1007/s12012-021-09659-w)
Supplement: Supplementary file 1 — Supplementary file1 (DOCX 354 kb) [file 12012_2021_9659_MOESM1_ESM.docx]

| ***Supplementary* Table 1. Variability of QT and QTc values measured from 12-lead Holter ECG at different phases of daily activity and sleep** | | | | | | | | | | | | | | | | | | | | | |
| --- | --- | --- | --- | --- | --- | --- | --- | --- | --- | --- | --- | --- | --- | --- | --- | --- | --- | --- | --- | --- | --- |
| **Date** | **Tx** | **[K+]** | **Diary** | **RR** | **QT** | **QTc B** | | | **QTc F** | **QTc H** | | | **Ventricular repolarization (12-lead Holter ECG)** | |  | |  | |  | |  |
| **April 15th, 2020** | Tamoxifen 20 mg/die | 3,3 mmol/L | moderate daily activity | 744 | 430,0 | 498,5 | | | 474,5 | 466,1 | | | Slightly scooped ST, U wave |  | |  | |  | |  |  |
|  |  |  | intense daily activity | 633 | 400,0 | 502,8 | | | 465,9 | 460,9 | | | ST↓, T wave flat /positive |  | |  | |  | |  |  |
|  |  |  | **90' after tamoxifen assumption** | 506 | 383,0 | 538,4 | | | 480,6 | 485,5 | | | ST↓, T wave negative V2-V5 |  | |  | |  | |  |  |
|  |  |  | mental work | 800 | 415,0 | 464,0 | | | 447,0 | 441,3 | | | Aspecific ST-T wave alteration, U wave |  | |  | |  | |  |  |
|  |  |  | cardiofitness | 479 | 370,0 | 534,6 | | | 472,9 | 484,2 | | | Minor ascending ST↓, T wave positive |  | |  | |  | |  |  |
|  |  |  | recovery | 860 | 428,0 | 461,5 | | | 450,1 | 445,1 | | | Aspecific ST-T alteration, U wave |  | |  | |  | |  |  |
|  |  |  | NREM sleep | 1306 | 506,0 | 442,8 | | | 462,9 | 481,4 | | | Aspecific ST-T alteration, U wave |  | |  | |  | |  |  |
|  |  |  | REM sleep | 678 | 467,0 | 567,2 | | | 531,6 | 516,9 | | | T-wave negative V1-V4 |  | |  | |  | |  |  |
|  |  |  | Wake-up & levothyroxine assumption | 572 | 450,0 | 595,0 | | | 542,1 | 528,6 | | | ST↓, T negative V2-V5 |  | |  | |  | |  |  |
|  |  |  | after few minutes | 880 | 415,0 | 442,4 | | | 433,1 | 429,3 | | | T wave negative V1-V5 |  | |  | |  | |  |  |
|  |  |  | intense daily activity | 667 | 444,0 | 543,7 | | | 508,2 | 496,4 | | | ST↓, T negative V2-V4 |  | |  | |  | |  |  |
| **April 24th, 2020** | Tamoxifen 20 mg/die | 3,7 mmol/L | moderate daily activity | 744 | 390,0 | 452,1 | | | 430,4 | 426,1 | | | normal |  | |  | |  | |  |  |
|  |  |  | intense daily activity | 633 | 395,0 | 496,5 | | | 460,0 | 455,9 | | | Slightly scooped ST, U wave |  | |  | |  | |  |  |
|  |  |  | **90' after tamoxifen assumption** | 622 | 415,0 | 526,2 | | | 486,2 | 478,8 | | | Slightly scooped ST, U wave |  | |  | |  | |  |  |
|  |  |  | mental work | 795 | 415,0 | 465,4 | | | 448,0 | 442,1 | | | normal |  | |  | |  | |  |  |
|  |  |  | cardiofitness | 570 | 380,0 | 503,3 | | | 458,3 | 459,2 | | | T wave: negative V1-V5, flat inferior & V6 |  | |  | |  | |  |  |
|  |  |  | recovery | 850 | 425,0 | 461,0 | | | 448,7 | 443,5 | | | normal |  | |  | |  | |  |  |
|  |  |  | NREM sleep | 1460 | 540,0 | 446,9 | | | 476,0 | 506,9 | | | T wave negative/flat V1-V4 |  | |  | |  | |  |  |
|  |  |  | REM sleep | 740 | 460,0 | 534,7 | | | 508,6 | 496,9 | | | T wave negative V1-V5 |  | |  | |  | |  |  |
|  |  |  | Wake-up & levothyroxine assumption | 585 | 422,0 | 551,7 | | | 504,6 | 496,5 | | | ST↓, T wave negative V2-V5 |  | |  | |  | |  |  |
|  |  |  | after few minutes | 990 | 444,0 | 446,2 | | | 445,5 | 445,1 | | | Aspecific T wave alteration |  | |  | |  | |  |  |
|  |  |  | intense daily activity | 650 | 422,0 | 523,4 | | | 487,2 | 478,5 | | | ST↓, T negative V2-V5 |  | |  | |  | |  |  |
| **May 7th, 2020** | no Tamoxifen | 3,3 mmol/L | moderate daily activity | 760 | 405,0 | 464,6 | | | 443,8 | 438,2 | | | Aspecific T wave alteration, U wave |  | |  | |  | |  |  |
|  |  |  | intense daily activity | 624 | 380,0 | 481,1 | | | 444,7 | 443,3 | | | Slightly scooped ST, U wave |  | |  | |  | |  |  |
|  |  |  | **no tamoxifen** | 770 | 422,0 | 480,9 | | | 460,4 | 453,4 | | | U-wave |  | |  | |  | |  |  |
|  |  |  | mental work | 839 | 433,0 | 472,7 | | | 459,1 | 453,1 | | | U-wave |  | |  | |  | |  |  |
|  |  |  | cardiofitness | 544 | 350,0 | 474,5 | | | 428,7 | 438,0 | | | Minor ascending ST↓, T wave positive |  | |  | |  | |  |  |
|  |  |  | recovery | 810 | 410,0 | 455,6 | | | 439,8 | 434,6 | | | T wave positive U wave |  | |  | |  | |  |  |
|  |  |  | NREM sleep | 1289 | 495,0 | 436,0 | | | 454,8 | 471,5 | | | T wave positive U wave |  | |  | |  | |  |  |
|  |  |  | REM sleep | 678 | 430,0 | 522,2 | | | 489,5 | 479,9 | | | T wave negative V1-V5 |  | |  | |  | |  |  |
|  |  |  | Wake-up & levothyroxine assumption | 670 | 428,0 | 522,9 | | | 489,1 | 479,7 | | | ST↓, T wave negative V2-V5 |  | |  | |  | |  |  |
|  |  |  | after few minutes | 833 | 405,0 | 443,7 | | | 430,4 | 426,1 | | | Minor ST↓, T wave negative V2-V6 |  | |  | |  | |  |  |
|  |  |  | intense daily activity | 683 | 406,0 | 491,3 | | | 461,0 | 454,7 | | | ST↓, T negative V2-V5 |  | |  | |  | |  |  |
| **October 8th, 2020** | Exemestane 25 mg/die | 3,8 mmol/L | moderate daily activity | 744 | 378,0 | 438,2 | | | 417,2 | 414,1 | | | Slightly scooped ST, U wave |  | |  | |  | |  |  |
|  |  |  | intense daily activity | 640 | 390,0 | 487,5 | | | 452,6 | 449,1 | | | Minor ascending ST↓, T wave positive |  | |  | |  | |  |  |
|  |  |  | **90' after exemestane assumption** | 628 | 383,0 | 483,3 | | | 447,2 | 445,2 | | | normal |  | |  | |  | |  |  |
|  |  |  | mental work | 795 | 390,0 | 437,4 | | | 421,0 | 417,1 | | | normal |  | |  | |  | |  |  |
|  |  |  | cardiofitness | 495 | 330,0 | | 469,0 | 417,2 | | | 437,1 | Minor ascending ST↓, T wave positive | |  | |  | |  | |  |  |
|  |  |  | recovery | 722 | 383,0 | | 450,7 | 426,9 | | | 423,4 | normal | |  | |  | |  | |  |  |
|  |  |  | NREM sleep | 1260 | 470,0 | | 418,7 | 435,2 | | | 448,3 | normal | |  | |  | |  | |  |  |
|  |  |  | REM sleep | 700 | 415,0 | | 496,0 | 467,4 | | | 460,0 | T wave negative V1-V5 | |  | |  | |  | |  |  |
|  |  |  | Wake-up & levothyroxine assumption | 645 | 394,0 | | 490,6 | 456,0 | | | 451,8 | T wave negative V1-V5 | |  | |  | |  | |  |  |
|  |  |  | after few minutes | 878 | 425,0 | | 453,6 | 443,8 | | | 439,6 | T negative V1-V5 (minor) | |  | |  | |  | |  |  |
|  |  |  | intense daily activity | 717 | 417,0 | | 492,5 | 465,9 | | | 458,4 | Aspecific ST alteration V2-V5 | |  | |  | |  | |  |  |

| ***Supplementary* Table 2. Average QT and QTc values measured from 12-lead Holter ECG** | | | | | | | | | |
| --- | --- | --- | --- | --- | --- | --- | --- | --- | --- |
| **A** | **April 15th, 2020** | | **April 24th, 2020** | | **May 7th, 2020** | | **October 8th, 2020** | |  |
|  | **Mean** | **SD** | **Mean** | **SD** | **Mean** | **SD** | **Mean** | **SD** |  |
| **RR** | 738,6 | 230,1 | 785,4 | 256,9 | 772,7 | 194,5 | 747,6 | 196,4 |  |
| **QT** | 428,0 | 38,5 | 428,0 | 43,7 | 414,9 | 35,9 | 397,7 | 34,9 |  |
| **QTc B** | 508,3 | 51,7 | 491,6 | 38,8 | 476,9 | 27,9 | 465,2 | 26,8 |  |
| **QTc F** | 479,0 | 34,7 | 468,5 | 25,7 | 454,7 | 20,5 | 440,9 | 18,7 |  |
| **QTc H** | 476,0 | 31,2 | 466,3 | 26,7 | 452,0 | 18,4 | 440,4 | 15,9 |  |
|  | **Tamoxifen 20 mg/die** | | | | **No drug** | | **Exemestane 25 mg/die** | |  |
|  | **[K+] 3,3 mmol/L** | | **[K+] 3,7 mmol/L** | | **[K+] 3,3 mmol/L** | | **[K+] 3,8 mml/L** | |  |

| **B** | **April 15th, 2020** | | **May 7th, 2020** | |  |  | **C** | **April 24th, 2020** | | **October 8th, 2020** | |  |
| --- | --- | --- | --- | --- | --- | --- | --- | --- | --- | --- | --- | --- |
|  | **Mean** | **SD** | **Mean** | **SD** | *p* |  |  | **Mean** | **SD** | **Mean** | **SD** | *p* |
| **RR** | 739,0 | 230,0 | 773,0 | 194,0 | *n.s.* |  | **RR** | 785,4 | 256,9 | 747,6 | 196,4 | *n.s.* |
| **QT** | 428,0 | 38,5 | 414,9 | 35,9 | *n.s.* |  | **QT** | 428,0 | 43,7 | 397,7 | 34,9 | *n.s.* |
| **QTc B** | 508,3 | 51,7 | 476,9 | 27,9 | *n.s.* |  | **QTc B** | 491,6 | 38,8 | 465,2 | 26,8 | *n.s.* |
| **QTc F** | 479,0 | 34,7 | 454,7 | 20,5 | *n.s.* |  | **QTc F** | 468,5 | 25,7 | 440,9 | 18,7 | *< 0,05* |
| **QTc H** | 476,0 | 31,2 | 452,0 | 18,4 | *< 0,05* |  | **QTc H** | 466,3 | 26,7 | 440,4 | 15,9 | *< 0,05* |
|  | **Tamoxifen 20 mg/die** | | **No drug** | |  |  |  | **Tamoxifen 20 mg/die** | | **Exemestane 25 mg/die** | |  |
|  | **[K+] 3,3 mmol/L** | | | |  |  |  | **[K+] 3,7-3,8 mmol/L** | | | |  |

n.s. : *not significant*

***
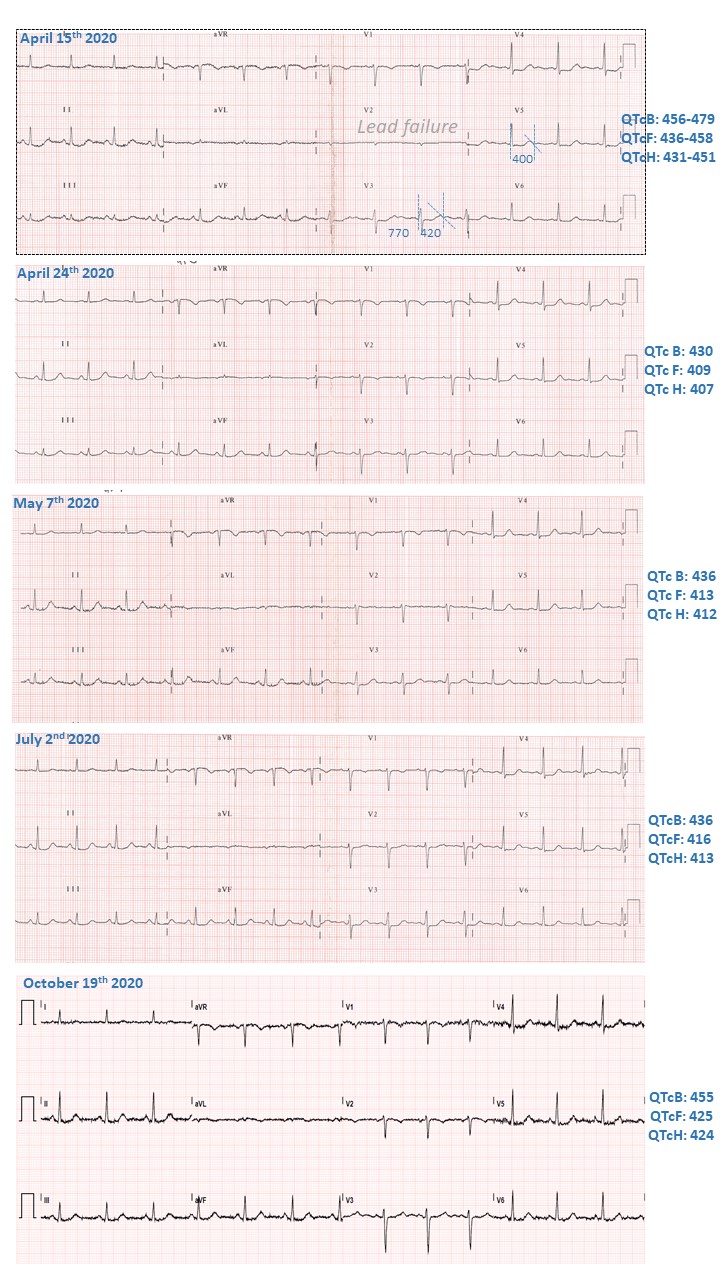
***

***Supplementary* Figure 1.** Sequence of standard 12-lead ECG, recorded during each ambulatory control, with no evidence of QTc interval prolongation. Only aspecific aspecific ventricular repolarization are appreciable.

***
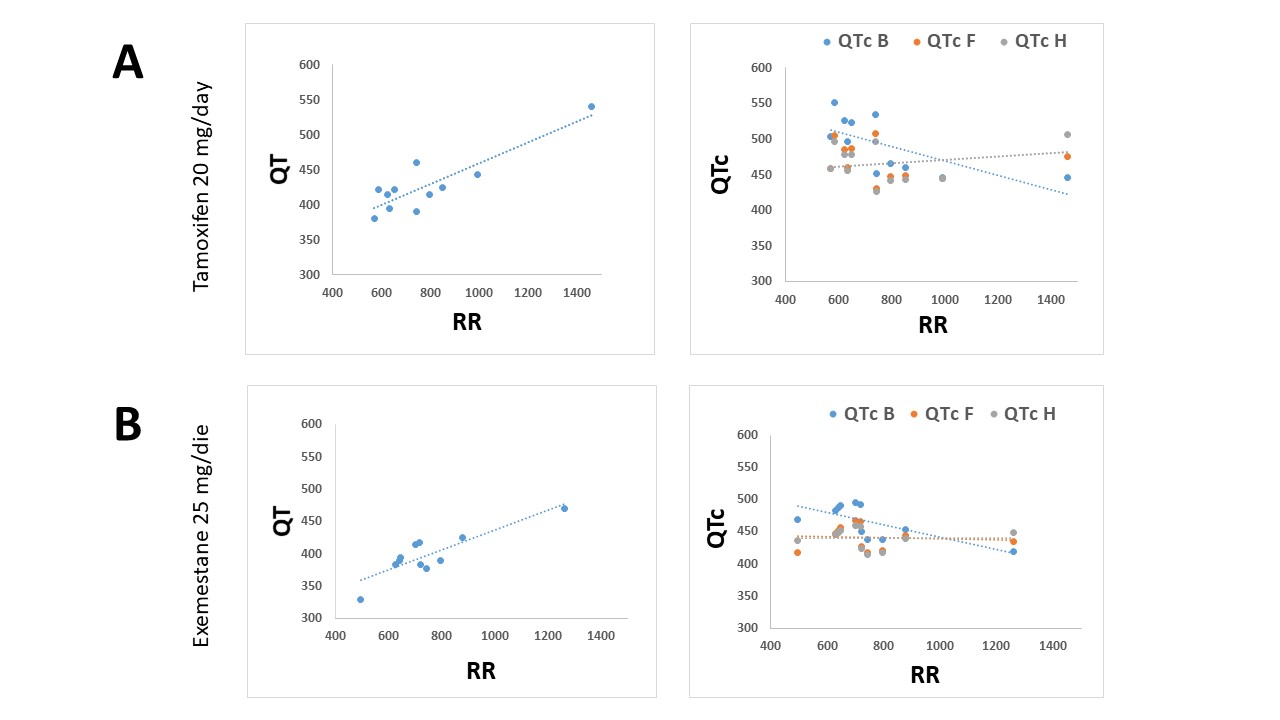
Supplementary* Figure 2.** Representative examples of the relationship between QT/QTc and RR, at the selected phases of daily activity and night sleep (see also the
supplementary table 1), during tamoxifen **(A)** and exemestane **(B)** treatments.
At higher heart rates, the Bazett’ s formula overestimated the QTc compared with the Fridericia and Hodges ones. However, under sympathetic activation *(refer to the supplementary Table 1)* also the QTc H values were abnormal (> 470 msec and even exceeding 500 msec) under tamoxifen treatment **(A)**, and normalized when tamoxifen was substituted with exemestane **(B)**
